# Genetic Testing in Adults over 50 Years with Chronic Kidney Disease: Diagnostic Yield and Clinical Implications in a Specialized Kidney Genetics Clinic

**DOI:** 10.3390/genes16040408

**Published:** 2025-03-31

**Authors:** Clara Schott, Mohammad Alajmi, Mohammad Bukhari, Sydney Relouw, Jian Wang, Adam D. McIntyre, Cadence Baker, Samantha Colaiacovo, Carla Campagnolo, Gabriela Almada Offerni, Peter G. Blake, Micheal Chiu, Andrea Cowan, Amit X. Garg, Lakshman Gunaratnam, Andrew A. House, Shih-Han Susan Huang, Hariharan Iyer, Arsh K. Jain, Anthony M. Jevnikar, John Johnson, Khaled Lotfy, Louise Moist, Faisal Rehman, Pavel S. Roshanov, Nabil Sultan, Matthew A. Weir, Pari Basharat, Anita Florendo-Cumbermack, Tayyab Khan, Jenny Thain, Kendrah Kidd, Stanislav Kmoch, Anthony J. Bleyer, Jaspreet Bhangu, Robert A. Hegele, Dervla M. Connaughton

**Affiliations:** 1 Department of Biochemistry, Schulich School of Medicine & Dentistry, Western University, 1151 Richmond St., London, ON N6A 5C1, Canada; clara.schott@lhsc.on.ca; 2 Department of Medicine, Division of Nephrology, London Health Sciences Centre, London, ON N6A 5A5, Canada; 3 Robarts Research Institute, Schulich School of Medicine and Dentistry, Western University, 1151 Richmond St., London, ON N6A 3K7, Canada; 4 Division of Medical Genetics, Department of Pediatrics, Victoria Hospital, London Health Science Center, 800 Commissioners Rd E, London, ON N6A 5W9, Canada; 5 Department of Medicine, Division of Nephrology, Schulich School of Medicine & Dentistry, Western University, 1151 Richmond St., London, ON N6A 5C1, Canada; 6 Multi-Organ Transplant Program, London Health Sciences Centre, London, ON N6A 5A5, Canada; 7 Department of Epidemiology and Biostatistics, Schulich School of Medicine & Dentistry, Western University, 1151 Richmond St., London, ON N6A 5C1, Canada; 8 Population Health Research Institute, 20 Copeland Avenue, Hamilton, ON L8L 2X2, Canada; 9 Department of Medicine, Division of Rheumatology, Schulich School of Medicine & Dentistry, Western University, 1151 Richmond St., London, ON N6A 5C1, Canada; 10 Department of Clinical Neurological Sciences, Schulich School of Medicine & Dentistry, Western University, 1151 Richmond St., London, ON N6A 5C1, Canada; 11 Department of Endocrinology and Metabolism, Sciences, Schulich School of Medicine & Dentistry, Western University, 1151 Richmond St., London, ON N6A 5C1, Canada; 12 Department of Medicine, Division of Geriatric Medicine, Sciences, Schulich School of Medicine & Dentistry, Western University, 1151 Richmond St., London, ON N6A 5C1, Canada; 13 Section on Nephrology, Wake Forest School of Medicine, Winston-Salem, NC 27157, USA; 14 Research Unit for Rare Diseases, Department of Pediatrics and Adolescent Medicine, First Faculty of Medicine, Charles University, CZ-121 08 Prague, Czech Republic

**Keywords:** chronic kidney disease, genetic kidney disease, genetic testing, older adults, exome sequencing

## Abstract

**Background:** Genetic causes of chronic diseases, once considered rare in adult-onset disease, now account for between 10 and 20% of cases of chronic kidney disease (CKD). Confirming a genetic diagnosis can influence disease management; however, the utility of genetic testing in older adults remains poorly understood, partly due to age-based restrictions on testing access. To better evaluate the diagnostic yield and clinical utility of genetic testing in this population, we analyzed data from adults aged ≥50 years with CKD who were assessed in a specialized kidney genetics clinic. **Methods:** We studied a cohort of 125 adults with CKD aged ≥50 years at the time of genetic testing. Genetic testing included gene panels targeting disease-related genes based on clinical phenotype, and/or exome sequencing for additional monogenic causes if the initial panel testing was inconclusive. **Results:** Pathogenic variants in disease-related genes were identified in 38% of patients. The highest diagnostic yield (48%) was in patients aged 50–54 years. The most common diagnosis post-testing was glomerulopathies (32%). Clinical utility, shown through the case series, included modifications to treatment and clinical management, as well as a reduction in the diagnostic odyssey. **Conclusions:** Our findings from a dedicated Kidney Genetics Clinic show that genetic testing in adults ≥50 years with CKD has significant diagnostic and clinical utility. These results support guideline recommendations that there should be no upper age limit for genetic testing. Future research in unselected CKD populations is needed to establish the broader applicability and feasibility of genetic testing in older adults.

## 1. Introduction

Genetic causes were once thought to be rare in patients with adult-onset chronic kidney disease (CKD). Data now indicate that between 10 and 20% of all CKD cases in adults are attributable to genetic causes [1,2]. This is not isolated to patients with CKD, with increasing recognition of monogenic contribution in other chronic diseases [3,4,5]. Additionally, in patients with genetic risk factors, such as a positive family history of CKD and the presence of multi-system disease, the prevalence of genetic kidney disease is even higher at 40% [6]. With the growing demand for the integration of genetic testing in medical specialties such as nephrology [7], clinical practice guidelines are being developed to assist healthcare providers in determining which patients should be offered genetic testing [8]. Some guidelines recommend restricting genetic testing based on age [9,10,11,12,13]; while others advocate for no upper age limit [14,15]. This inconsistency may contribute to the limited use of genetic testing in older adults. Age-based restrictions and lack of access likely stem from the long-standing assumption that the genetic contribution to chronic diseases significantly diminishes with age [16]. For instance, the high prevalence of CKD in individuals 50 years of age and older is frequently attributed to comorbid conditions and environmental risk factors such as hypertension, diabetes mellitus, atherosclerotic cardiovascular disease, smoking, and previous kidney injury [17].

Recent studies have found pathogenic genetic variants in a substantial proportion of older patients with CKD, with one study yielding a diagnosis for 21% of participants that had a median age of 55 years at the time of genetic testing [1]. Furthermore, they demonstrated significant clinical benefits with over 90% of patients, who tested positive for genetic kidney disease, experiencing a change in their clinical management, and 34% having a direct change in treatment [1].

Herein, we determined the diagnostic yield of genetic testing in patients with CKD assessed in a kidney genetics clinic, who were 50 years or older at the time of genetic testing. We illustrate the clinical utility of these patients through several case descriptions.

## 2. Methods

We undertook a prospective cohort study of CKD patients encompassing all age categories, with previously reported methods [14]. In this study, we analyzed patients 50 years of age and older at the time of review in a Kidney Genetic Clinic in Southwestern Ontario, Canada (Western University Research Ethics Board #115160) [14]. We use the Kidney Disease Improving Global Outcomes (KDIGO) definition of CKD; kidney damage or glomerular filtration rate (eGFR) <60 mL/min/1.73 m^2^ for 3 months or more, irrespective of cause [18]. The time of CKD onset is defined as first diagnosis of CKD by a nephrologist. Informed consent was obtained from all participants. Clinical and pedigree data were collected using a standardized questionnaire, with additional information extracted from participants’ medical records. Race and ethnicity were self-reported.

**Figure 1:**
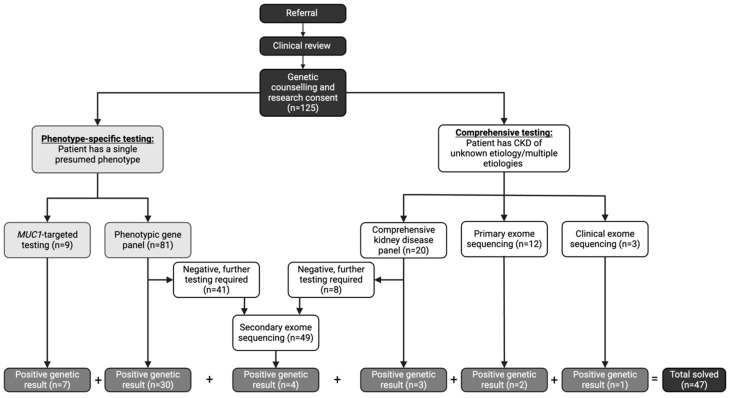
Genetic Testing Workflow in a Dedicated Kidney Genetics Clinic. CKD (chronic kidney disease). Referral; referral to the Kidney Genetics Clinic.

Genetic testing followed a stepwise approach based on the clinical suspicion of the underlying subtype of CKD (Figure 1). All participants underwent genetic testing using one of the following five approaches [14]:

(1) **Phenotypic-driven gene panel testing** which includes genes specific for a certain subtype of disease (i.e., cystic kidney disease). This is the first-line approach when a specific CKD subtype is suspected.
(2) **Comprehensive inherited kidney disease gene panel** which includes 331 genes known to cause CKD across different phenotypes. This approach is employed when the phenotype is ambiguous or multiple overlapping conditions are suspected.
(3) ***MUC1*-targeted gene testing** for the cytosine insertion in the *mucin-1* (*MUC1*) gene in participants with high clinical suspicion of autosomal dominant tubulo-interstitial kidney disease (ADTKD) (i.e., non-proteinuric CKD with onset ≥18 years, family pedigree consistent with an autosomal dominant mode of inheritance, bland urine sediment at initial diagnosis of CKD, and personal or family history of gout) [19]. This testing is not currently available on any commercial sequencing platforms; therefore, the testing was performed in the Clinical Laboratory Improvement Amendments (CLIA) certified laboratory at the Broad Institute and Massachusetts Institute of Technology. This targeted testing is required due to the high guanosine-cytosine content and repetitive nature of the 60-mer variable number of tandem repeat (VNTR) sequences [20].
(4) **Clinical exome sequencing** is employed as a first line test in eligible participants who met the Genome-Wide Sequencing Ontario criteria for testing which included the following: moderate to severe developmental or functional impairment, multisystem involvement, progressive clinical course, differential diagnosis required multiple targeted gene panels, and suspected severe genetic syndrome for which multiple family member are affected or where parents are consanguineous [21].
(5) **Research exome sequencing** assessing for all implicated causes of genetic CKD (694 genes) using a virtual, bioinformatic panel approach [22] in individuals who did not meet current clinical criteria for clinical exome sequencing (**primary**) or who remained undiagnosed after panel testing (**secondary**). This exome sequencing is performed in a research laboratory with findings confirmed in a CLIA certified laboratory through specific mutation confirmation, as previously described [14].

In some cases, additional phenotypes were observed at the time of assessment in the Kidney Genetics Clinic, that did not necessarily cause CKD but could impact kidney function or management and therefore additional genetic testing in non-CKD causing genes was performed on a case-by-case basis. Genetic results were reviewed by a multi-disciplinary team that included a physician with expertise in kidney genetics, a genetic counselor, and a research team skilled in genomic data and sequencing interpretation.

### 2.1. Primary Outcome

The primary outcome was “solved” status, which we defined as identification of pathogenic or likely pathogenic (P/LP) variant as defined by the American College of Medical Genetics guidelines [23], where the genotype was consistent with the observed disease phenotype.

### 2.2. Statistical Analysis

We calculated the diagnostic yield as a percentage of participants—out of all who were tested—who were genetically solved with a P/LP variant in a gene known to cause the disease in question. We reported both the participant (*n* = 125) and familial diagnostic yield (*n* = 114). A priori risk factor analysis included the age of ESKD and CKD onset, family history of CKD, and the presence of extrarenal features. The univariate Chi-squared test was used to determine *p* values for categorical variables. For categories with numbers less than 5 in any group, a two-tailed Fisher’s exact test was used. Logistic regression was performed to assess whether positive family history and age of onset of CKD and ESKD are associated with solved status, adjusting for age at testing and gender. *p*-values from logistic regression analyses were adjusted for age at genetic testing and sex using the Benjamini–Hochberg method to control for the false discovery rate.

### 2.3. Health Outcomes Related to Clinical Utility

Health outcome measures were captured post disclosure of genetic results and repeated at study close, across five predefined levels of clinical utility [14]. The pre-defined measures of clinical utility were adapted from a toolkit devised by Hayeems et al., which was developed using the Fryback and Thornbury model for evidence collection for clinical utility of genomics sequencing [24]. Health outcomes measures include (1) diagnostic accuracy, (2) diagnostic thinking efficacy, (3) therapeutic efficacy, (4) patients outcomes efficacy, and (5) societal efficacy.

### 2.4. Case Descriptions

To highlight the clinical utility of genetic testing in older adults, a detailed case series including participants with four genetic diagnoses are described. We detail the clinical history, genetic testing results, and impact on clinical management after genetic diagnosis.

## 3. Results

### 3.1. Demographics

Overall, 125 participants from 114 families, all ≥50 years at the time of genetic testing were included (Table 1). The cohort included an equal number of males and females, and most patients self-reported as white (64%, *n* = 88/125). The indication for genetic assessment was a combination of extrarenal features in 81% (*n* = 100/125) and a positive family history of CKD in 74% (*n* = 92/125). ESKD was established in 38% (*n* = 38/125), with 52% progressing to ESKD after 50 years of age. The most frequent diagnosis before genetic testing was CKD of unknown etiology (CKDu) (48%, *n* = 60/125). In total, 174 genetic tests (either gene panel or exome analysis) were performed, with phenotypic gene panels being the most common (47%, *n* = 81/125), followed by exome sequencing in 28% (*n* = 49/125).

### 3.2. Diagnostic Yield

**Figure 2:**
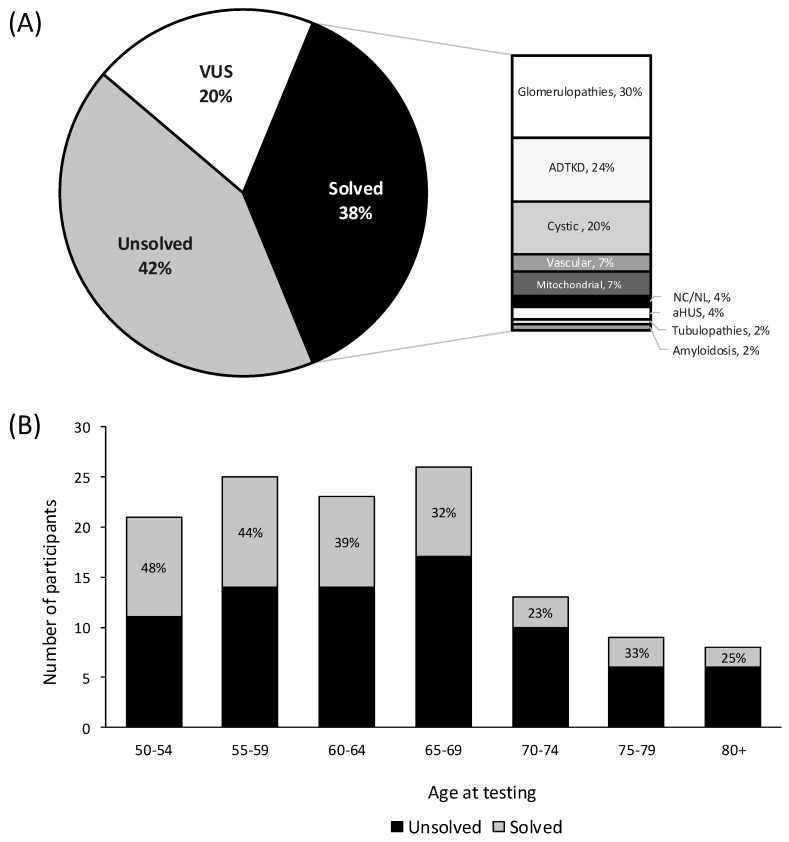
Diagnostic yield of chronic kidney disease patients ≥ 50 years of age at time of genetic testing. (**A**) Overall diagnostic yield (**B**) Diagnostic yield stratified by age at genetic testing. Participants are either solved with a pathogenic variant, which has definitive evidence of disease causation, or likely pathogenic which have strong but not absolute evidence (>90% certainty) and are both considered diagnostic in clinical genetics [23]. Unsolved participants can either have a VUS (variants of uncertain significance), or no identified variants. Abbreviations: atypical hemolytic uremic syndrome (aHUS), autosomal dominant tubulointerstitial kidney disease (ADTKD), and nephrocalcinosis and nephrolithiasis (NC/NL).

The diagnostic yield was 38% (*n* = 47/125), with 35% (*n* = 40/114) of families receiving a genetic diagnosis (Figure 2A, Table S1). The most common genetic diagnoses after testing were glomerulopathies including collagenopathies (32%, *n* = 14/47), ADTKD (24%, *n* = 11/47), and cystic kidney disease (20%, *n* = 9/47). Notably, 35% (*n* = 21/60) diagnosed with CKDu, prior to genetic testing, received a diagnosis after testing (Table 1). Stratifying by testing type, gene panels including comprehensive and phenotypic-driven gene panels had a diagnostic yield of 37% (*n* = 37/101). Exome sequencing including clinical (i.e., primary) and research (i.e., secondary) had a diagnostic yield of 11% (*n* = 7/64), and *MUC1*-specific testing had a yield of 78% (*n* = 7/9) (Table 1). Diagnostic yield was the highest in participants aged 50–54 years (48%, *n* = 10/21), although the yield remained high across all age groups (Figure 2B). P/LP variants were detected in 20 different genes in the 47 solved participants. Variants in the genes *MUC1* (*n* = 7/47), *COL4A3* (*n* = 6/47), *PKD1* (*n* = 5/47), *APOL1* (*n* = 4/47), and *UMOD* (*n* = 4/47) were most common (Table 2).

### 3.3. Risk Factors for Genetic Disease

A higher diagnostic yield was observed in those with a positive family history of CKD (43% versus 21%, *p* = 0.03 odds ratio (OR) 2.93 (1.19, 8.05)). Participants with a CKD onset after 50 years of age had a lower diagnostic yield (27% versus 50%, *p* = 0.06, OR 0.41 (0.16, 1.02) (Table S2)). When analyzed as a group, the presence of extrarenal features were not significantly associated with a genetic diagnosis (40% versus 34%, *p* = 0.65); however, when stratified by subtype of extra-renal features, certain conditions—such as hearing loss (73%, *p* = 0.02) and cystic liver disease (86%, *p* = 0.01)—were associated with a higher likelihood of a genetic diagnosis (Table 1). Recommendations based on risk factors for genetic kidney disease are shown in Table 3.

### 3.4. Time to Diagnosis

Despite all participants undergoing genetic testing ≥50 years, 66% (*n* = 31/47) of those with positive genetic testing results were diagnosed with CKD before the age of 50, with 34% (16/47) diagnosed with CKD at age 50 or older. The median time to a genetic diagnosis from initial CKD diagnosis was longer in those diagnosed with CKD before the age of 50, compared to participants diagnosed with CKD ≥50 years (24.7 ± 14.9 versus 8.6 ± 6.4 years).

### 3.5. Clinical Utility

**Figure 3:**
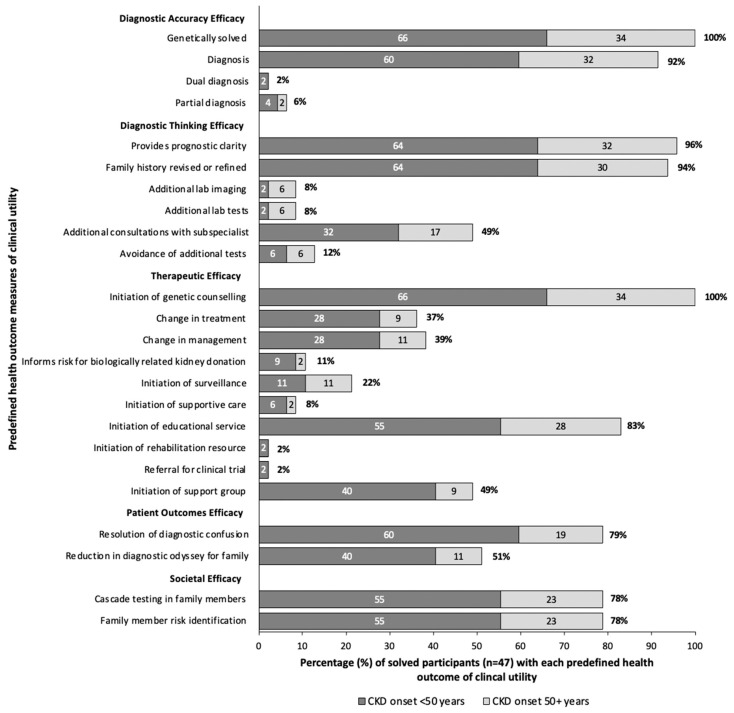
Clinical utility in solved participants (*n* = 47). Clinical utility is determined by predefined health outcomes, modified from Hayeems et al. [24]. The dark gray represents patients diagnosed with CKD before the age of 50, light gray represents those diagnosed at 50 years or older. The numbers within each bar represent the percent of solved patients with that health outcome for each age group, and the percent at the end of the bar represents the overall percentage of solved patients with the health outcome.

In our clinic, all 47 participants who received a genetic diagnosis received post-test genetic counseling. Assessing clinical utility, 96% gained prognostic clarity, 79% had resolution in diagnostic confusion, and 78% could avail of cascade testing for family members. Referral to subspecialists took place for 49% of participants, and 39% experienced a change in clinical management (Figure 3). Seventeen participants (37%) had a direct modification in treatment which included the initiation of angiotensin-converting enzyme inhibitors with Alport Disease, initiation of eculizumab peri-transplant for one participant with a variant in *CFH*, or initiation of disease specific treatments for participants with variants in *ADAMTS13*, *POLG*, and *PHEX* as discussed in the following case series. Overall, when stratifying by age of CKD onset, participants with onset of CKD <50 years, and those ≥50 years at time of CKD onset experienced similar clinical utility, proportional to the percentage genetically solved (Figure 3).

## 4. Case Descriptions

### 4.1. Case 1. X-Linked Hypophosphatemia

#### 4.1.1. Patient History

**Participant 626 (P626)** is a 72-year-old female with chronic hypophosphatemia and recurrent fractures, unresponsive to treatment. In childhood, she underwent corrective surgeries for genu varum and surgical interventions for bilateral distal radius and ankle fractures in early adulthood. She had a right total knee arthroplasty, surgical correction of a right tibial midshaft periprosthetic fracture and right femur intramedullary nail fixation following a pathologic midshaft femur fracture (Figure S1). On systems review she had hearing impairment, requiring bilateral hearing aids in her third decade. She reported no family history of kidney disease or bone disorders. At the time of assessment, serum creatinine was 88 µmol/L, corresponding to an estimated Chronic Kidney Disease Epidemiology Collaboration (CKD-EPI) Glomerular Filtration Rate (eGFR) of 57 mL/min/1.73 m^2^. Her vitamin D and calcium levels were within normal range. The main reason for referral to the Kidney Genetics clinic were low phosphate levels despite phosphate replacement ranging 0.60–0.80 mmol/L, with an elevated parathyroid hormone (PTH) level of 11.5 pmol/L. A urine dipstick test was negative for blood and protein.

#### 4.1.2. Genetic Testing, Diagnosis, and Pathophysiology

Following genetic assessment, a Bone Fracture and Fragility gene panel encompassing 74 genes identified a likely pathogenic, heterozygous, frameshift variant in the *PHEX* gene (NM_000444.6, c.1075_1076del, p.K359Qfs*20, ClinVar ID: VCV002631066.1) (Figure S1). Loss-of-function variants in *PHEX* cause X-linked hypophosphatemia (XLH) (OMIM# 307800), the most common phosphate metabolism disorder, with an estimated prevalence of 1 in 20,000–70,000 [25]. The *PHEX* gene encodes the phosphate-regulating endopeptidase, which plays a crucial role in regulating fibroblast growth factor 23 (FGF23) levels. FGF23, in turn, regulates serum inorganic phosphate concentrations. Loss-of-function variants, such as the one identified in P626, result in elevated levels of circulating FGF23, leading to renal phosphate wasting, phosphaturia, and hypophosphatemia [26]. Clinically, XLH manifests with a variety of skeletal abnormalities, including growth retardation, rickets, osteomalacia, bone pain, enthesopathy, osteoarthritis, spontaneous dental abscesses, hearing loss, and muscular dysfunction. Men typically experience more severe affects from the disease than women; however, women can have clinical manifestations, ranging from mild hypophosphatemia to progressive osteomalacia [27,28]. Recent studies show that kidney conditions are present in 24% of children and 11% of adults with XLH [25]. Notably, a study of 579 patients with XLH, including 65 participants aged ≥50 years, revealed that the mean time from symptom onset to genetic diagnosis was 9.4 years, but significantly longer for adults ≥50 years [25]. In this case, P626 was diagnosed with XLH at age 72, indicating a long diagnostic delay [28]. This highlights the need for improved awareness of inherited phosphate-wasting disorders in adults with unexplained hypophosphatemia.

#### 4.1.3. Clinical Implications and Management

Confirmation of the genetic disease had a direct impact on treatment, with the initiation of Burosomab therapy. Burosumab is a human monoclonal IgG1 antibody directed against FGF23 which can improve joint stiffness, physical function, fracture healing, and osteomalacia in adults with XLH [29]. This genetic diagnosis can also provide prognostic clarity, enabling subspecialist referral for management and the initiation of supportive care, including rehabilitation resources. Furthermore, genetic counseling and cascade testing can now be offered to at-risk biologically related family members.

### 4.2. Case 2. Mitochondrial Cytopathies

#### 4.2.1. Patient History

**Participant 779 (P779)** is a 67-year-old female referred for investigation of recurrent rhabdomyolysis of uncertain etiology. She was initially referred for assessment at the age of 60 with an elevated creatinine of 138 µmol/L. Although she had long-standing hypertension, she was noted to be hypotensive at initial review, prompting discontinuation of her antihypertensive medications. Additionally, she had a diagnosis of well-controlled diabetes mellitus (HbA1C 4.8%), managed with diet and metformin, with no evidence of diabetic retinopathy or neuropathy. Subsequently, she presented with an acute episode of pneumonia and septic shock, leading to acute kidney injury (AKI) that required the initiation of hemodialysis. After discharge, she had partial kidney recovery with creatinine stabilizing at 140 µmol/L, consistent with stage 3 CKD. The cause of her CKD was subsequently attributed to a combination of type 2 diabetes mellitus, hypertension, confounded by AKI. Over time, she had several hospital admissions for generalized muscle weakness and falls. During one admission, an elevated creatine kinase (CK) level of 853 IU/L was noted, attributed to statin therapy for hypercholesterolemia, prompting discontinuation. However, she had another acute episode of rhabdomyolysis with no obvious precipitant, marked by 3 days of progressive malaise, weakness, and dark-colored urine. During this episode, her creatinine rose to 554 µmol/L, with CK peaking at 22,000 IU/L (Table S3A). Systems review revealed bilateral hearing impairment, onset in the third decade, requiring bilateral hearing aids by the age of 60. Collateral history from family members describes progressive ptosis and a history of diplopia, at the end of the day or after periods of fine motor work. Over recent months, she reported progressive unsteadiness in her gait, now requiring a broad stance to maintain balance. Nerve conduction studies were normal, but electromyography (EMG) revealed myopathic changes, with evidence of muscle membrane irritability, primarily affecting the proximal muscles, especially in the lower extremities. Based on these findings, a working diagnosis of myositis was made, and she was started on steroid therapy while awaiting a muscle biopsy.

#### 4.2.2. Genetic Testing and Diagnosis

Following genetic assessment, a Metabolic Myopathies, Rhabdomyolysis, and Exercise Intolerance Panel, which included 83 genes, revealed a pathogenic variant c.1399G>A, p.A467T (GenBank ID: NM_002693.3) in the *POLG* gene (Table S3B). This gene encodes DNA polymerase gamma, an enzyme critical for mitochondrial DNA replication. The A467T variant detected in P779, as previously reported, with functional data demonstrating a significant reduction in polymerase γ activity to 4% of wild-type levels, impairing oxidative phosphorylation and mitochondrial function in a dominant manner [30,31]. Although disease can occur in the recessive state in children and young adults, heterozygous variants in *POLG*, including A467T, are associated with autosomal dominant Progressive External Ophthalmoplegia (PEO) (OMIM #174763), with typical onset > 40 years. Despite the name PEO, these mitochondrial cytopathies are multisystem disorders that present with overlapping phenotypes [30,32]. In this case, P779 presented with characteristic symptoms of ptosis, progressive external ophthalmoplegia, ataxia, and muscle weakness [33].

Renal involvement has been reported in approximately 11% of patients with *POLG*-related disease [33], with reported renal manifestations including tubulopathies, renal tubular acidosis, nephrocalcinosis, and CKD of unclear etiology. In this case, multiple factors could have contributed to the development of CKD, including diabetes, hypertension, and prior episodes of rhabdomyolysis-induced AKI. However, her HbA1c was well-controlled at 4.8%, and her blood pressure remained normal throughout follow-up without the need for antihypertensive therapy, underscoring the potential over-reliance on attributing CKD to diabetes and hypertensive nephropathy.

#### 4.2.3. Clinical Implications and Management

The confirmation of a *POLG* variant provided clarity regarding the etiology of these recurrent clinical presentations and resolved diagnostic uncertainty. It negated the need for invasive diagnostic procedures, including muscle biopsy. Retrospective analysis suggests that earlier genetic testing could have avoided EMG testing. Along with avoidance of steroid treatment, Table 4 outlines additional treatment precautions that should now be considered.

### 4.3. Cases 3 and 4. Atypical Cystic Kidney Disease

#### 4.3.1. Patient Histories

**Participant 518 (P518)** is a 70-year-old male referred for genetic testing to investigate polycystic kidney disease (PKD). He was diagnosed with CKD at age 65, following an incidental finding of an elevated creatinine level of 154 µmol/L. Initially, CKD was attributed to hypertension and type 2 diabetes mellitus. However, an ultrasound revealed bilaterally enlarged kidneys with numerous cysts (Figure S2). He progressed to ESKD at age 68 and is currently on hemodialysis. During genetic consultation, P518 reported a positive family history of CKD in his father, who despite having polycystic kidney disease (PKD), did not progress to kidney failure during his lifetime. A 9-gene cystic kidney disease panel revealed a pathogenic, heterozygous, frameshift variant c.1867_1870del p.Glu623Argfs*20 in *IFT140* (GenBank ID: NM_014714.3), confirming a diagnosis of cystic kidney disease.

**Participant 22 (P22)** is a 61-year-old male who was referred for genetic testing with an assumed diagnosis of PKD. He first presented to nephrology at age 57 with flank pain and hypertension. Abdominal imaging revealed non-alcoholic fatty liver disease and multiple bilateral kidney cysts (Figure S3). At the time of genetic assessment, his creatinine was normal at 90 µmol/L. He too reported a positive family history of non-progressive cystic kidney disease in his mother and brother. A 9-gene cystic kidney disease panel revealed a pathogenic heterozygous splice site variant c.1359_1359+3delinsAC in *IFT140* (GenBank ID: NM_014714.3), again confirming a diagnosis of cystic kidney disease.

#### 4.3.2. Genetic Insights into Cystic Kidney Disease

ADPKD is the most common hereditary cystic kidney disease, with an incidence of 1 in 1000–2500 individuals [34]. It is primarily associated with *PKD1* (~78%) and *PKD2* (~15%) leading to bilateral kidney cysts, progressive enlargement, and a decline in kidney function over time. Affected individuals are also at increased risk for intracranial aneurysms and polycystic liver disease [34]. Approximately 7% of cystic kidney disease cases-sometimes termed atypical cystic kidney disease, are linked to variants in genes, such as *GANAB*, *DNAJB11*, *HNF1B*, *PRKCSH*, *SEC63*, *ALG8*, *ALG9*, *SEC61B*, *LRP5*, and *SEC61A1A* [34]. Notably, *IFT140* variants, traditionally associated with autosomal recessive ciliopathies, have been recently implicated in 4.5% of autosomal dominant cystic kidney disease cases [35,36]. Individuals with heterozygous loss-of-function *IFT140* variants present with large kidney cysts, fewer liver cysts, and a milder disease course with slower progression.

#### 4.3.3. Clinical Implications and Importance of Genetic Diagnosis

We highlight two cases initially suspected as PKD, later confirmed by genetic testing as *IFT140*-related cystic kidney disease. Compared to *PKD1* or *PKD2* variants, *IFT140* carriers typically have smaller kidney volumes, less liver involvement, and a lower risk of ESKD, though progression remains possible, particularly with comorbidities like hypertension or diabetes. Recognizing this variability is critical for personalized treatment, tailored surveillance strategies, and genetic counseling. These cases also underscore the risk of misdiagnosis and highlight the necessity of genetic testing before attributing a clinical diagnosis to *PKD1* or *PKD2* variants alone.

### 4.4. Case 5. Congenital Thrombotic Thrombocytopenia Purpura

#### 4.4.1. Patient History

**Participant 577 (P577)** is a 61-year-old male with a history of congenital anomalies of the kidney and urinary tract (CAKUT). Imaging in early adulthood revealed a solitary kidney, and he progressed to ESKD, requiring dialysis at age 59. He also experienced asymptomatic thrombocytopenia, with platelet counts ranging from 30 to 110 × 10⁹/L, and had a stroke in his 50’s. At age 61, he underwent a successful kidney transplantation achieving a creatinine of 88 µmol/L within one week. However, post-operatively, his platelet count dropped from 104 × 10^9^/L pre-transplant to 38 × 10^9^/L accompanied by active bleeding and a hemoglobin decline to 67 g/L. Investigations at the time revealed a positive hemolytic workup (Table S4A) and critically low ADAMTS13 levels (<4%) which led to a working diagnosis of Thrombotic Thrombocytopenic Purpura (TTP). Initial genetic testing for complement-mediated kidney disease identified a VUS in the *CFI* gene (associated with Atypical Hemolytic Uremic Syndrome), prompting further testing. Comprehensive genetic testing identified a homozygous pathogenic *ADAMTS13* variant (c.3178C>T, p.Arg1060Trp) (Table S4B), confirming a diagnosis of congenital TTP. While this did not clarify the cause of his CKD (i.e., single kidney), it explained his thrombocytopenia and possible progression in CKD, guiding peri-transplant management.

TTP is a rare, life-threatening microangiopathic hemolytic anemia characterized by thrombocytopenia, hemolytic anemia, renal dysfunction, and neurologic symptoms [37]. It results from severe ADAMTS13 deficiency, leading to uncontrolled platelet aggregation and microthrombi formation, causing end-organ damage [37]. The disease is rare, with an incidence of 1.5–6 cases per million and typically present in mid-life. Recessive *ADAMTS13* variants account for 5–8% of TTP cases [37]. Diagnosing TTP can be challenging, due to overlapping symptoms with other thrombotic microangiopathies, contributing to delayed or misdiagnosis. The morbidity associated with TTP is significant, with neurological complications, AKI, and bleeding complications leading to a high mortality rate [37]. Indeed repeated microthrombotic episodes could have contributed to progressive renal injury due to recurrent endothelial damage from clinical or subclinical episodes [38].

#### 4.4.2. Clinical Implications

In P577, genetic testing confirmed congenital TTP only after transplantation, despite the history of recurrent thrombocytopenia and stroke both suggestive of TTP. This delay in diagnosis may have stemmed from attributing thrombocytopenia to other CKD-related causes [39], and lack of routine genetic testing in kidney transplant candidates. Earlier genetic diagnosis could have influenced transplant management, particularly in balancing immunosuppression with thrombotic risk. This case highlights the under-utilization of genetic testing in CKD patients. Once TTP was diagnosed, regular plasma infusions—the mainstay of treatment—were initiated, significantly improving outcomes [37]. This case also emphasizes the importance of comprehensive genetic testing, especially when initial results are inconclusive.

### 4.5. Autosomal Dominant Tubulo-Interstitial Kidney Disease

Herein, we highlight the cases of seven patients from three families, all of whom tested positive for a pathogenic variant in the *MUC1* gene, consistent with the diagnosis of ADTKD (OMIM#174000) (Table 5). ADTKD is a group of genetic disorders characterized by autosomal dominant inheritance, bland urine (absence of blood or protein on urine dipstick), and progressive CKD, with a median age of progression to ESKD of 48 years [40]. Variants in several genes including *MUC1*, *UMOD*, *REN*, and more recently *APOA4*, are known to cause the disease [40,41]. ADTKD-*MUC1* is caused by pathogenic variants in the *MUC1* gene, typically a frameshift variant, most often due to a cytosine insertion within the Variable Number Tandem Repeat (VNTR) region [40]. Testing for variants in *MUC1* is an ongoing challenge due to the high guanine/cytosine content of the VNTR region (>80%), which makes it difficult to detect using standard sequencing platforms.

#### Diagnostic Odyssey in ADTKD

These cases illustrate the challenges in diagnosing ADTKD-*MUC1* and the potential for misdiagnosis to other subtypes of CKD. Due to the lack of specific symptoms and the challenges of testing, ADTKD-*MUC1* remains difficult to diagnose and clinicians must maintain a high level of suspicion when evaluating these cases [40]. The patients in this series were all referred due to a positive family history of CKD. The most common pre-testing diagnosis was CKDu; however, others were misdiagnosed with cystic kidney disease or hereditary nephritis, with both inter- and intra-familial variation in the reported cause of CKD. Most had already undergone a long diagnostic odyssey by the time genetic assessment was considered, with a median time from the first diagnosis of CKD to the genetic confirmation of 22.1 ± 7.35 years. It is worth noting the finding that all participants had progressed to ESKD prior to consideration of genetic testing. This suggests systematic delays in genetic diagnosis potentially due to limited clinical awareness, misclassification of disease etiology, and historical barriers to genetic testing access [42,43]. For families with ADTKD-*MUC1*, genetic testing is the gold standard for diagnosis, providing diagnostic clarity and negating the need for invasive, non-diagnostic procedures such as kidney biopsies. Early genetic testing in these families could have informed prognosis, guided management, and facilitated family screening. Establishing a genetic diagnosis in these families is also important for future generations, as drug treatment trials with new targeted therapies, such as BRD4780, are currently being developed as potential treatment targets for ADTKD-*MUC1* and could benefit future generations of affected families [44].

## 5. Discussion

This study provides valuable insights into the utility of genetic testing in adults ≥50 years following assessment in a Kidney Genetics Clinic. We demonstrate a 38% diagnostic yield after genetic testing, which aligns with previously reported data, demonstrating a median diagnostic yield of 40% in adults with CKD [6]. Studies examining genetic testing in more unselected CKD cohorts have reported diagnostic yields ranging from 10% to 20% [1,2], suggesting that our higher yield likely reflects the selective nature of evaluating patients with suspected genetic disease. Larger studies will be necessary to determine the true prevalence of monogenic CKD in all adults over 50.

One major barrier to the implementation of genetic testing in older adults is the lack of clear guidance on who should undergo testing [45]. Our findings, in line with previous reports [46,47,48], demonstrate that a positive family history of CKD is a significant risk factor for genetic disease. Other factors include multi-system disease and extra-renal manifestations [46]. Although our study is limited by a small sample size, we observe that cystic liver disease and hearing loss before the age of 50 are associated with genetic kidney disease in our cohort.

Nearly half of the cohort had CKD of unknown etiology prior to genetic testing, with 36% receiving a genetic diagnosis following testing. The most frequent diagnoses after testing included glomerulopathies including collagenopathies, ADTKD, and cystic kidney disease, along with rarer genetic kidney disorders. Given that over 500 single-gene causes of CKD have been identified—many occurring at frequencies of <1% in study populations [6], comprehensive genetic testing is increasingly necessary to ensure accurate diagnoses and tailored treatment strategies. In addition, while some identified variants (e.g., P779; *POLG*, P577; *PHEX*) are not classified as primary kidney disease genes, their detection had significant clinical implications, including avoiding nephrotoxic medications, guiding peri-transplant care, and informing family genetic counseling.

Multiple barriers impede genetic testing in older adults, including the historic exclusion from clinical genomic studies [49]. This has contributed to limited access to genetic testing for patients aged ≥50 years, particularly in nephrology [10,12,50,51]. Furthermore, 80% of nephrologists report either “limited” or “no prior education in genetics”, underscoring the persistent under-recognition of genetic disease [43]. Consequently, the diagnostic odyssey remains protracted for many patients with GKD. This is especially evident for patients with ADTKD, where the median time from the initial CKD diagnosis to the genetic confirmation is 22 years. ADTKD typically presents with non-specific clinical and kidney biopsy features, making genetic testing the gold standard for definitive diagnosis [19]. Given that disease onset can range from 16 to 80 years [52], our findings reinforce the clinical value of genetic testing across all age groups. Our study highlights diagnostic delays, particularly among individuals diagnosed with CKD before age 50 years who experience a median delay of 24 years compared to 8 years for those diagnosed ≥50 years. This discrepancy likely reflects historical barriers in genetic testing, reliance on biochemical and histopathological assessments, and low clinical suspicion for genetic disease, with CKD often attributed to acquired or environmental factors such as hypertension, diabetes, or glomerulonephritis [6,43]. In contrast, increased awareness, and improved access to testing in recent years have facilitated earlier diagnoses in recent years [14] even in older adults with CKD. The establishment of the Kidney Genetics Clinic in 2020 further enhanced access to testing, whereas genetic evaluation was previously less accessible for adults with CKD. This likely introduced a temporal bias contributing to prolonged diagnostic delays observed. Older adults in our cohort may have benefited from genetic testing earlier in their diagnostic journey due to greater clinical awareness and accessibility, whereas younger individuals diagnosed with CKD in earlier decades faced longer delays due to historical barriers to genetic testing.

Despite growing recognition of genetic testing in CKD [15], systemic barriers—including cost, limited clinical awareness, and the absence of standardized testing protocols—continue to hinder accessibility [53]. Expanding governmental and insurance coverage to reduce out-of-pocket costs, along with enhanced genetic education for nephrologists and primary care providers, could improve test utilization [42,43]. Additionally, the establishment of standardized guidelines for when and how to perform genetic testing could facilitate earlier diagnosis and optimize patient management.

A common counterargument against genetic testing in older adults is its perceived lack of clinical utility beyond merely “naming” the disease. However, our findings refute this. We demonstrate nearly 40% of patients experience direct changes to their management and treatment following a confirmed genetic diagnosis. While the diagnostic yield is higher in individuals with younger CKD onset (50% if onset CKD <50 years versus 27% in those with CKD onset ≥50 years) [1], we find that clinical utility persists across all age groups.

This study provides key insights into genetic testing in older adults but has limitations. The modest sample size (125 participants) reduces the statistical power of subgroup analyses. Selection bias is also a concern, as recruitment from a specialized Kidney Genetics Clinic likely reflects a subset of CKD patients with a higher genetic disease probability, limiting generalizability. Additionally, some identified variants were in genes not traditionally linked to CKD, potentially inflating diagnostic yield, though they still had clinical relevance for patients with CKD. Larger, multicenter studies of unselected CKD patients aged ≥50 years are needed to determine the true prevalence of GKD. Future research should also assess evolving diagnostic timelines, cost-effectiveness, and broader integration of genetic testing into general nephrology to optimize healthcare utilization.

## 6. Conclusions

Our findings support genetic testing in older adults with CKD, demonstrating a high diagnostic yield and significant clinical utility, including impacts on treatment decisions, disease management, and prognostication. These results highlight the need to eliminate age-based restrictions for genetic testing in eligible CKD patients, ensuring broader access to genetic evaluation and personalized care. As an essential diagnostic tool, genetic testing enhances disease classification and enables tailored treatment strategies for appropriately selected CKD patients.

## Figures and Tables

**Table 1:** Demographics of CKD patients over the age of 50.

	Total Participants (*n* (Proportion in %))	Solved Participants (*n*)	Unsolved Participants (*n*)	Diagnostic Yield (%)	*p* Value
**Total participants**	125	47	78	38	
**Total families**	114	40	74	35	
**Sex**					
Male	59 (47)	20	39	33	0.53
Female	66 (53)	27	39	41	
**Age at testing**					
50–54	21 (17)	10	11	48	0.43
55–59	25 (20)	11	14	44	0.61
60–64	23 (18)	9	14	39	1.00
65–69	26 (21)	9	17	35	0.11
70–74	13 (10)	3	10	23	0.37 †
75+	17 (14)	5	12	29	0.63
**Race**					
Asian	8 (7)	0	8	0	0.02 †*
Black	6 (5)	4	2	67	0.20 †
Hispanic	3 (2)	3	0	100	0.05 †*
Indigenous	4 (3)	0	4	0	0.17 †
White	80 (64)	34	46	43	0.19
Unknown	24 (19)	6	18	25	0.24
**Age at CKD diagnosis**					
<18	7 (6)	3	4	42	1.00 †
19–29	15 (12)	4	11	22	0.41 †
30–39	18 (14)	14	4	78	0.0002 †*
40–49	22 (18)	10	12	46	0.55
50–59	28 (24)	8	20	29	0.37
60+	32 (26)	8	24	25	0.14
Unknown	3 (2)	0	3	0	0.29 †
**CKD onset (known)**					
50+ years	60 (49)	16	44	27	0.06 ^◊^
<50 years	62 (51)	31	31	50	
**ESKD**					
Present	48 (38)	23	25	48	0.14 ^◊◊^
Absent	77 (62)	24	53	31	
**Age at ESKD onset**					
50+ years	25 (52)	10	15	40	0.39
<50 years	23 (48)	13	10	57	
**CKD etiology**					
Unknown	61 (49)	22	39	36	0.86
Presumed	64 (51)	25	39	39	
**Etiology if presumed**					
ADTKD	1 (1)	1	0	100	0.39 †
CAKUT	5 (8)	1	4	20	0.64 †
Cystic kidney disease	22 (34)	11	11	50	0.30
Diabetic nephropathy	7 (11)	2	5	29	0.70 †
Glomerulopathies	12 (19)	4	8	33	0.75 †
Hypertensive nephropathy	10 (16)	4	6	40	1.00 †
Stones disease	5 (8)	2	3	40	1.00 †
Tubulopathies	2 (3)	0	2	0	0.52 †
**Family history**					
Positive	92 (74)	40	52	43	0.03 *^◊◊◊^
Negative	33 (26)	7	26	21	
**Extrarenal Features**					
Positive	78 (62)	31	47	40	0.65
Negative	47 (38)	16	31	34	
**Type of extrarenal feature**					
Hearing loss	25 (32)	13	12	52	0.20
*Onset < 50 years*	11 (48)	8	3	73	0.02 †*
*Onset ≥ 50 years*	13 (52)	5	8	38	0.84
Gout	35 (45)	11	24	31	0.26
*Proceeding CKD*	12 (34)	4	8	33	0.75 †
*Following CKD*	23 (66)	7	16	30	0.41
Valvular heart disease	1 (1)	0	1	0	1.00 †
Hypospadias	1 (1)	0	1	0	1.00 †
Neurological disorder	6 (8)	2	4	33	1.00 †
Liver disease	13 (17)	8	5	62	0.15
*Cystic liver*	7 (54)	6	1	86	0.01 †*
*Fatty liver*	5 (39)	2	3	40	1.00 †
*Other*	1 (8)	0	1	0	1.00 †
Eye pathology	28 (36)	8	20	29	0.20
**Testing type**					
Comprehensive gene panel	20 (12)	3	17	15	0.29 †
Phenotypic gene panel	81 (47)	30	51	37	0.009 *
Clinical exome	3 (2)	1	2	33	1.00 †
Primary exome	12 (7)	2	10	17	0.52 †
Secondary exome	49 (28)	4	45	8	0.0005 †*
*MUC1*-specific testing	9 (5)	7	2	78	0.002 †*

**Footnote:** † Two-tailed Fisher’s exact test. * Statistical significance *p* = < 0.05. Adjusted *p*-values using Benjamini–Hochberg method for sex and gender: ^◊^ CKD age 50+ = 0.09, ^◊◊^ ESKD = 0.14, ^◊◊◊^ positive family history = 0.08. Definitions: age at CKD diagnosis is the initial contact with medical services which confirmed CKD; age at testing is the age at which the patients received genetic testing; a comprehensive gene panel includes 331 genes known to cause CKD through Prevention Genetics (code 13990); the phenotypic gene panel tests a group of genes for a specific kidney disease phenotype; clinical exome sequencing is exome sequencing performed in a clinical laboratory; primary exome sequencing is exome sequencing performed as a first-line test in a research laboratory; secondary exome sequencing is exome sequencing performed as a second-line test for patients who had a negative gene panel, it is also a research test; *MUC1*-specific testing sequences the variable tandem repeat region (VNTR) region of *MUC1* gene. Extrarenal features: eye pathology includes blindness, cataracts, corrective eye disease, glaucoma, macular degeneration, and retinal detachment; neurological manifestations include developmental delay and epilepsy. Testing type: total of 174 tests for 125 participants, therefore the testing type is shown as a proportion of total tests (174). Abbreviations: Chronic kidney disease (CKD), congenital anomalies of the kidney and urinary tract (CAKUT), end stage kidney disease (ESKD).

**Table 2:** Genotypes and phenotypes of solved patients (*n* = 47).

Phenotype	Genotype	Percent of Solved Patients
Glomerulopathies including collagenopathies (14)	*COL4A3* (6), *APOL1* (3), *COL4A5* (3), *COL4A4* (2)	30%
Tubulointerstitial kidney disease (11)	*MUC1* (7), *UMOD* (4)	24%
Cystic kidney disease (9)	*PKD1* (5), *IFT140* (2), *ALG5* (1), *PKD2* (1)	20%
Mitochondrial and Rhabdomyolysis (4)	*POLG* (2), *CPT2* (1), *MTTL1* (1)	9%
Vascular kidney disease including hypertension (3)	*ABCC6* (3)	7%
Nephrocalcinosis and nephrolithiasis (2)	*SLC2A9* (1), *SLC3A1* (1)	4%
Complement/aHUS and other (2)	*ADAMTS13* (1), *CFH* (1)	4%
Tubulopathies (1)	*PHEX* (1)	2%
Amyloidosis (1)	*FGA* (1)	2%

**Footnote**: Number in brackets represents the number of patients within each phenotype or genotype.

**Table 3:** Recommendations for genetic testing in the general chronic kidney disease population.

Feature	Diagnostic Yield in Our Study	Recommendation
Early-onset CKD (<50 years)	50%	Strongly consider testing
Family history of CKD	43%	Strongly consider testing
Extrarenal features	40%	Consider testing
CKD of unknown etiology	36%	Consider testing

**Footnote:** CKD (chronic kidney disease).

**Table 4:** Recommendations for management of mitochondrial diseases including *POLG*-related disorders.

Clinical Outcome	Specific Outcome
Avoidance of certain medications	Acetaminophen: chronic or frequent use may deplete glutathione and cause hepatopathy. Aminoglycosides: may cause hearing loss. Antiretrovirals: may worsen peripheral neuropathy, liver dysfunction or myopathy. Botulism toxin: may worsen weakness. Metformin and topiramate: may cause lactic acidosis. Statins: statin therapy should be approached with caution due to risk of exacerbating myopathy. If initiated, close monitoring of CK levels and symptoms of muscle weakness are required. Valproic acid: may cause irreversible fulminant liver failure and should never be administered.
Referral to subspecialists for extrarenal a feature of disease	Neurological for assessment of risk of Parkinsonism, cognitive impairment, mood disorders, and sleep disorders. Physiotherapy assessment to assess motor skills and mobility. Occupational therapy for assessment of use of adaptive devices over time. Hepatology for monitoring of abnormal liver blood test. Ophthalmology review to assess reduced vision, abnormal ocular movement, best corrected visual acuity, refractive errors, strabismus, cataracts, and retinal dystrophy.
Use of additional lab images	MRI of brain. USS of liver.
Surveillance	Renal functional test at 1–2-year intervals.

**Footnote:** modified from Parikh et al. 2017 [32]. Abbreviations: creatinine kinase (CK), magnetic resonance imaging (MRI), and ultrasound scan (USS). While these represent general recommendations for the management of mitochondrial disease, treatment must be tailored to the specific needs of each patient.

**Table 5:** Patients diagnosed with *MUC1*-ADTKD.

Family #	Patient #	Presumed Etiology Pre-Testing	Age CKD/ESKD Onset Years	Age at MUC1 Diagnosis Years	Family History	Extrarenal Features	Hematuria, Proteinuria	Genetic Testing Strategy
F0006	P0007	Atypical cystic kidney disease	48/54	57	+	Macular degeneration	Hematuria	Cystic kidney disease panel, 2° exome sequencing, *MUC1*-targeted testing
	P0306	ADTKD	34/60	63	+	Gout	Bland urine	*MUC1*-targeted testing
F0022	P0027	CKDu	31/43	55	+	Hyperpara-thyroidism	Bland urine	FSGS and HHL panels *, *MUC1*-targeted testing
	P0050	Hereditary nephritis	38/43	65	+	Hearing loss, hypertension	Bland urine	FSGS and HHL panels, *MUC1*-targeted testing
F0303	P0442	CKDu	42/53	62	+	None	Hematuria, proteinuria	*MUC1*-targeted testing
	P0504	CKDu	30/42	59	+	None	Proteinuria	*MUC1*-targeted testing
	P0516	CKDu	37/37	54	+	None	Bland urine	*MUC1*-targeted testing

**Footnote:** All patients are positive for a cytosine duplication in the variable number tandem repeat (VNTR) domain of *MUC1*. (*) testing was completed prior to clinic review. Abbreviations: CKDu (chronic kidney disease of unknown etiology), FSGS (focal segmented glomerulosclerosis), HHL (hereditary hearing loss), *MUC1* (Mucin-1), number (#), positive (+), and 2° (secondary).

## Data Availability

Additional data can be shared by contacting the senior author upon request.

## Supplementary Materials

The following supporting information can be downloaded at: https://www.mdpi.com/article/10.3390/genes16040408/s1, Figure S1: Patient 626 Clinical and Genetic Findings. Figure S2. Patient 518 Clinical and Genetic Findings. Figure S3. Patient 22 Clinical and Genetic Findings. Table S1. Patients ≥ 50 years solved with pathogenic/likely pathogenic variants in genes known to cause chronic kidney disease (n = 47) [54,55]. Table S2. Logistic Regression Analysis of Patients with a Genetic Diagnosis. Table S3. Patient 779 Clinical Laboratory and Genetic Findings. Table S4. Patient 577 Clinical Laboratory and Genetic Findings.
