# Supplementary material for: Genetic Testing in Adults over 50 Years with Chronic Kidney Disease: Diagnostic Yield and Clinical Implications in a Specialized Kidney Genetics Clinic
Source: Genes (Basel). 2025 Mar 31;16(4):408. doi: 10.3390/genes16040408 (PMC12027001; doi:10.3390/genes16040408)
Supplement: Supplementary file 1 [file genes-16-00408-s001.zip › genes-3515821-supplementary.pdf]

Supplementary Material for Genetic Testing in Adults Over 50 years with Chronic Kidney Disease: Diagnostic Yield and Clinical Implications in a Specialized Kidney Genetics Clinic.

This file outlines individual patient and case data relevant for the manuscript.

Supplementary Tables:

Table S1. Patients ≥50 years solved with pathogenic/likely pathogenic variants in genes known to cause chronic kidney disease (n=47).

Family No.	Patient No./ Sex	Age CKD /ESKD onset	Family History	Extrarenal Features	*Pre-priori* diagnosis	Test used to confirm genetic diagnosis	Gene	Zygosity and Variant (HGVS description)	ACMG Class. (Criteria met)	gnom AD freq.	Post-priori diagnosis (OMIM#)
F0002 (15)	P0002/ F	30/NA	+	None	CKDu with proteinuria and microscopic hematuria	Alport Gene Panel	*COL4A5*	Heterozygous (mat.) Hemizygous (son) NM_033380.3:c.1217G>A NP_203699.1:p.(G406D)	Likely Pathogenic (PM1, PP2, PM2, PM5, PP3, PP5)	0	AD Alport Disease (mat.) X-linked Alport Syndrome (son) (#301050)
F0006 (15)	P0007/ F	48/54	+	Macular degeneration	Atypical Cystic Kidney Disease	*MUC1* specific gene testing	*MUC1*	Heterozygous cytosine duplication in VNTR region of *MUC1* gene	Pathogenic (PVS1, PS3, PP5)	NA	ADTKD (#17400)
	P0306/ M	34/60	+	Gout	ADTKD						
F0017 (15)	P0022/ M	53/NA	+	Non-alcoholic fatty liver disease	Cystic Kidney Disease	Cystic Kidney Disease Panel	*IFT140*	Heterozygous splicing variant NM_014714.4:c.1359_1359+3delinsAC	Likely Pathogenic (PVS1, PM2, PP5)	0	Atypical cystic kidney disease (*614620)
F0019 (15)	P0041/ F	37/NA	+	None	CKDu with hematuria	Familial variant testing	*COL4A3*	Heterozygous NM_000091.5:c.1372G>A NP_000082.2:p.(G458R)	Pathogenic (PS1, PM5, PM2, PM1, PP3, PP5)	0	Dominant Alport Syndrome (#104200) Autosomal

Family No.	Patient No./ Sex	Age CKD /ESKD onset	Family History	Extrarenal Features	*Pre-priori* diagnosis	Test used to confirm genetic diagnosis	Gene	Zygosity and Variant (HGVS description)	ACMG Class. (Criteria met)	gnom AD freq.	Post-priori diagnosis (OMIM#)
F0022 (15)	P0027/ F	31/43	+	Hyperparathy roidism	CKDu	*MUC1* specific gene testing	*MUC1*	Heterozygous cytosine duplication in VNTR region of *MUC1* gene	Pathogenic (PVS1, PS3, PP5)	NA	ADTKD (#17400)
F0035 (15)	P0050/ F	38/43	+	Hearing loss	Hereditary Nephritis	*MUC1* specific gene testing	*MUC1*	Heterozygous cytosine duplication in VNTR region of *MUC1* gene	Pathogenic (PVS1, PS3, PP5)	NA	ADTKD (#17400)
F0041 (15)	P0058/ F	33/45	+	Gout	FSGS	NS/ FSGS panel	*APOL1*	Homozygous G1 risk alleles in *APOL1* NM_003661.4:c.1024A>G NP_003652.2:p.(S342G); NM_003661.4:c.1152T>G NP_003652.2:p.(I384M)	Risk allele	0.0160, 0.0155	*APOL1* related kidney disease (#612551)
	P0077 M	38/38	+	None	Hyper-tensive nephropathy						
F0048 (15)	P0071/ F	46/46	+	Recurrent venous and arterial thrombosis, early onset stroke, recurrent miscarriage, and early kidney transplant loss	CKDu	Clinical ES (Due to extra-renal features)	*CFH*	Heterozygous NM_000186.4:c.504C>G NP_000177.2:p.(Y168*)	Pathogenic (PVS1, PM2)	NA *	Susceptibility to aHUS 1 (#235400)
F0049 (15)	P0128/ F	60/NA	+	Hypertension	CKDu	2° ES	*UMOD*	Heterozygous NM_003361.4:c.566A>G NP_003352.2:p.(Y189C)	Likely Pathogenic (PM1, PP2, PM2, PP3)	NA *	ADTKD (#162000)

Family No.	Patient No./ Sex	Age CKD /ESKD onset	Family History	Extrarenal Features	*Pre-priori* diagnosis	Test used to confirm genetic diagnosis	Gene	Zygosity and Variant (HGVS description)	ACMG Class. (Criteria met)	gnom AD freq.	Post-priori diagnosis (OMIM#)
F0051 (15)	P0074/ F	39/40	+	Gout	CKDu	Targeted gene panel with familial variant testing	*UMOD*	Heterozygous NM_003361.4:c.202G>A NP_003352.2:p.(E68K)	Likely Pathogenic (PM1, PP2, PM2, PM5, PP3)	NA *	ADTKD (#162000)
	P0075/ M	40/NA	+	Liver cysts, joint pain	CKDu						
	P0076/ M	20/52	+	Fatty liver disease, joint pain	CKDu						
F0060 (15)	P0092/ F	74/NA	+	Nephrolithiasis	Cystic kidney disease	Cystic kidney disease	*PKD1*	Heterozygous NM_001009944.3:c.3437_3439del NP_001009944.3:p.(F1146del)	Likely Pathogenic (PM4, PM5, PM2)	NA *	ADPKD (#173900)
F0063 (55)	P0096/ M	28/29	+	Skin manifestations, hypertension	Alport Syndrome	2° ES	*ABCC6*	Heterozygous NM_001171.6:c.3421C>T NP_001162.5:p.(R1141*)	Pathogenic (PM3, PVS1, PM2)	0.0014	Pseudoxanthoma elasticum (#177850)
F0073 (55)	P0050/ M	69/NA	+	Eyelid laxity	Hypertensive and diabetic nephropathy	1° ES	*ABCC6*	Heterozygous NM_001171.6:c.1999del NP_001162.5:p.(A667Qfs*21)	Pathogenic (PS4, PVS1, PM2)	NA *	Pseudoxanthoma elasticum (#177850)
	P0123/ M	40/41	+	Bilateral BKA							
F0078 (15)	P0115/ F	31/60	+	Gout, hearing loss, detached retina and lens dislocation	CKDu	Alport Panel	*COL4A3*	Compound Heterozygous NM_000091.5:c.2083G>A NP_000082.2:p.(G695R); NM_000091.5:c.4466C>T NP_000082.2:p.(T1489I)	Pathogenic (PM3, PP1, PP3, PM2), VUS (PM2, PP3)	0.0002, 0	Autosomal Recessive Alport Syndrome (#620536)
F0090 (15)	P0129/ M	42/43	-	None	CKDu	NS/FSGS Panel	*APOL1*	Homozygous G1 risk allele in *APOL1*	Risk allele	0.0161, 0.0155	*APOL1* related kidney disease (#612551)

Family No.	Patient No./ Sex	Age CKD /ESKD onset	Family History	Extrarenal Features	*Pre-priori* diagnosis	Test used to confirm genetic diagnosis	Gene	Zygosity and Variant (HGVS description)	ACMG Class. (Criteria met)	gnom AD freq.	Post-priori diagnosis (OMIM#)
								NM_003661.4:c.1024A>G NP_003652.2:p.(S342G); NM_003661.4:c.1152T>G NP_003652.2:p.(I384M)			
F0154 (15)	P0217/ F	2/23	+	Liver cysts, hearing loss, gout at age 10 years	MPGN	2° ES (Negative NS/FSGS panel)	*CPT2*	Heterozygous NM_000098.3:c.338C>T NP_000089.1:p.(S113L)	Pathogenic (PM2, PP3, PP5)	0.0016	CPT II deficiency, myopathic, stress-induced (#255110)
F0181	P0253/ M	47/48	+	Type 2 Diabetes Mellitus	Diabetic nephropathy	2° ES	*POLG*	Heterozygous NM_002693.3:c.2890C>T NP_002684.1:p.(R964C)	Pathogenic (PS4, PP3, PP2)	0.0007	POLG associated disorders (#157640)
F0191 (15)	P0264/ M	68/NA	+	Gout, type 2 diabetes, hypertension, cataracts, hearing loss	Stones Disease	NLNC panel	*SLC3A1*	Heterozygous NM_000341.4:c.1400T>C NP_000332.2:p.(M467T)	Likely Pathogenic (PM1, PP2, PM2, PM5 PP3, PP5)	0.0025	Cystinuria (#220100) Partial diagnosis
F0197 (15)	P0276/ F	73/NA	+	Diabetes, hearing loss, strokes, epilepsy	MELAS syndrome	MELAS testing	*MTTL1*	5% heteroplasmy NC_012920.1:m.3243A>G	Likely Pathogenic	NA *	MELAS syndrome (*590050)
F0202 (56)	P0283/ F	49/NA	+	Eye Pathology	CKDu	Compre-hensive KD panel	*COL4A4*	Heterozygous NM_000092.5:c.3307G>A NP_000083.3:p.(G1103R)	Likely Pathogenic (PS4, PP3, PM2)	0	Alport Syndrome (#141200)
F0205 (15)	P0537/ F	20/NA	+	Hearing loss	Collagenopathy	Alport panel	*COL4A5*	Heterozygous NM_033380.3:c.2767G>A NP_203699.1:p.(G923S)	Likely Pathogenic (PP3, PM2, PP2)	NA *	X-linked Alport Syndrome (#301050)
F0217	P0404/ M	32/NA	+	Gout	CKDu Hematuria Proteinuria	Alport Panel	*COL4A4*	Heterozygous NM_000092.5:c.4724C>	Likely Pathogenic	0.000004	Autosomal dominant Alport

Family No.	Patient No./ Sex	Age CKD /ESKD onset	Family History	Extrarenal Features	*Pre-priori* diagnosis	Test used to confirm genetic diagnosis	Gene	Zygosity and Variant (HGVS description)	ACMG Class. (Criteria met)	gnom AD freq.	Post-priori diagnosis (OMIM#)
								A NP_000083.3:p.(A1575E)	(PM2, PP1, PP3, PP4)		Syndrome (#141200)
F0224 (15)	P0317/ M	53/65	+	Cystic liver disease	PKD	Cystic kidney disease Panel	*PKD1*	Heterozygous NM_001009944.3:c.1198 C>T NP_001009944.3:p.(R400*)	Pathogenic (PVS1, PM2, PS2, PP5)	0	ADPKD (#173900)
F0228 (15)	P0522/ M	47/50	+	Hearing loss, Gout, cardiomyopathy, knee and ankle arthritis	Vasculitis	Compre-hensive KD panel	*SLC2A9*	Heterozygous splice site variant NM_020041.3:c.681+1G >T	Pathogenic (PVS1, PM2, PP5), partial diagnosis	0	Hypouricemia, renal, 2 (#612076) Partial diagnosis
F0254	P0373/ M	61/NA	+	Cataracts, liver cysts	PKD, atrophic polycystic kidneys progressive after 60 years	ADPKD Panel	*ALG5*	Heterozygous NM_013338.5:c.634C>T NP_037470.1:p.(R212C)	Likely Pathogenic (PM2, PM5, PP3, PP5, PP4)	0	Polycystic kidney disease 7 (#620056)
F0256 (15)	P0377/ F	51/NA	+	None	PKD	Cystic kidney disease panel	*PKD1*	Heterozygous intronic variant NM_001009944.3:c.9202 -16G>A	Likely Pathogenic (PS4, PM2)	0	ADPKD (#173900)
F0271 (15)	P0397/ F	57/63	-	None	PKD	Cystic kidney disease panel	*PKD1*	Heterozygous NM_001009944.3:c.303_305del NP_001009944.3:p.(N101del)	Likely Pathogenic (PS4, PM4, PM5, PM2)	NA *	ADPKD (#173900)
F0283 (15)	P0417/ M	70/NA	-	None	Amyloidosis with CKD	Amyloid panel	*FGA*	Heterozygous NM_021871.4:c.1634A>T NP_068657.1:p.(E545V)	Likely Pathogenic (PM2, PP5)	0	Amyloidosis, familial, visceral (#105200)
F0288 (15)	P0422/ F	54/NA	+	None	CKDu	Alport panel	*COL4A5*	Heterozygous NM_033380.3:c.187G>A NP_203699.1:p.(G63R)	Likely Pathogenic (PP3, PM2, PP2)	NA *	X-linked Alport Syndrome (#301050)

Family No.	Patient No./ Sex	Age CKD /ESKD onset	Family History	Extrarenal Features	*Pre-priori* diagnosis	Test used to confirm genetic diagnosis	Gene	Zygosity and Variant (HGVS description)	ACMG Class. (Criteria met)	gnom AD freq.	Post-priori diagnosis (OMIM#)
F0298 (15)	P0433/ F	29/NA	-	Hearing loss, gout	Glomerulonephritis	Compre-hensive KD panel	*COL4A3*	Heterozygous NM_000091.5:c.2567G>T NP_000082.2:p.(G856V)	Likely Pathogenic (PM2, PP3, PM1)	NA *	Autosomal dominant Alport Syndrome (#104200)
F0303 (15)	P0442/ F	42/53	+	None	CKDu	*MUC1* specific gene testing	*MUC1*	Heterozygous cytosine duplication in VNTR region of *MUC1* gene	Pathogenic (PVS1, PS3, PP5)	NA	ADTKD (#17400)
	P0504/ F	30/42									
	P0516/ F	37/37									
F0312 (15)	P0452/ F	47/NA	+	Gout, hypertension	Glomerulonephritis	NS/ FSGS panel	*COL4A3*	Heterozygous NM_000091.5:c.2452G>A NP_000082.2:p.(G818R)	Pathogenic (PM3, PP3, PM2)	0	Autosomal Dominant Alport Syndrome (#104200)
F0324 (15)	P0471/ M	39/NA	-	Hearing loss	CKDu	NS/FSGS panel	*COL4A3*	Heterozygous NM_000091.5:c.1622G>ANP_000082.2:p.(G541D)	Likely Pathogenic (PP3, PM2), VUS (BP4, PP5), VUS (BP4, PP5, BP6)	NA *	Autosomal Dominant Alport Syndrome (#104200)
							APOL1	Homozygous G1 risk allele NM_003661.4:c.1024A>G NP_003652.2:p.(S342G)	Risk allele	0.0161, 0.0155	APOL1 related kidney disease (#612551)
F0362 (15)	P0518/ M	64/68	+	Liver cysts, corrective eye disease	Cystic kidney disease	Cystic Kidney Disease panel	*IFT140*	Heterozygous NM_014714.4:c.1867_1870del NP_055529.2:p.(Glu623ArgfsTer20)	Pathogenic (PVS1, PM2, PP4)	0.000004	Atypical cystic kidney disease (*614620)

**Family No.**	**Patient No./ Sex**	**Age CKD /ESKD onset**	**Family History**	**Extrarenal Features**	***Pre-priori* diagnosis**	**Test used to confirm genetic diagnosis**	**Gene**	**Zygosity and Variant (HGVS description)**	**ACMG Class. (Criteria met)**	**gnomAD freq.**	**Post-priori diagnosis (OMIM#)**
F0368 (15)	P0525/ F	52/NA	+	Gout	Thin Basement Membrane Disease	Alport panel	*COL4A3*	Heterozygous NM_000091.5:c.1219G>C NP_000082.2:p.(G407R)	Likely Pathogenic (PM3, PM2, PP3)	NA *	Autosomal Dominant Alport Syndrome (#104200)
F0396 (15)	P0577/ M	56/56	-	Stroke, TIA, hypothyroidism with thrombocytopenia with low ADATS13 level post kidney transplant	CAKUT, single kidney	TTP panel	*ADAMTS13*	Homozygous NM_139027.6:c.3178C>T NP_620596.2:p.(R1060W)	Pathogenic (PM3, PP1, PS3, PM2)	0.0008	Thrombotic thrombocytopenic purpura, hereditary (#274150) Partial diagnosis
F4032 (15)	P0626/ F	72/NA	-	Recurrent fractures since childhood, rickets, chronic hypophosphatemia, hypertension	Hypertension Chronic Hypophosphatemia	Bone fragility and fracture panel	*PHEX*	Heterozygous NM_000444.6:c.1075_1076del NP_000435.3:p.(K359Efs*20)	Likely Pathogenic (PVS1, PM2, PP5)	NA	Hypophosphatemic rickets, X-linked (#307800)
F0443 (15)	P0627/ F	40/NA	+	Liver cysts, Oncocytoma kidney Hypertension Recurrent UTI	ADPKD	Cystic kidney disease panel	*PKD1*	Heterozygous NM_001009944.3:c.3719_3721del NP_001009944.3:p.(N1240del)	Likely Pathogenic (PS4, PP1, PM2, PM4)	NA *	ADPKD (#173900)
F0463	P0666/ M	59/NA	+	Liver cysts, bilateral hearing loss, bilateral	ADPKD	Cystic kidney disease panel	*PKD2*	Heterozygous NM_000297.4:c.2508C>G NP_000288.1:p.(Y836*)	Pathogenic (PS4, PVS1, PM2)	0.00088	ADPKD (#613095)

Family No.	Patient No./ Sex	Age CKD /ESKD onset	Family History	Extrarenal Features	*Pre-priori* diagnosis	Test used to confirm genetic diagnosis	Gene	Zygosity and Variant (HGVS description)	ACMG Class. (Criteria met)	gnom AD freq.	Post-priori diagnosis (OMIM#)
				retinal detachment							
F0543	P0779/ F	18/19	-	Fatty liver	CKDu	Rhabdomy olysis panel	*POLG*	Heterozygous NM_001126131.2: c.1399G>A NP_001119603.1: p.(A467T)	Pathogenic (PM3, PP1, PS3, PM2, PP3, PM5, PP2)	0.0005	POLG associated disorders (#157640)

Footnote: Abbreviations: ACMG criteria: pathogenic supporting 1 (PP1), pathogenic supporting 1 (PP2), pathogenic supporting 3 (PP3), pathogenic supporting 4 (PP4), pathogenic supporting 5 (PP5), pathogenic moderate 1 (PM1), pathogenic moderate 2 (PM2), pathogenic moderate 3 (PM3), pathogenic moderate 4 (PM4), pathogenic moderate 5 (PM5), pathogenic moderate 6 (PM6), pathogenic strong 1 (PS1), pathogenic strong 2 (PS2), pathogenic strong 3 (PS3), pathogenic strong 4 (PS4), pathogenic very strong (PVS1). Amino acids: alanine (A), arginine (R), asparagine (N), aspartic acid (D), cysteine (C), glutamic acid (E), glutamine (Q), glycine (G), histidine (H), Isoleucine (I), leucine (L), lysine (K), methionine (M), phenylalanine (F), proline (P), serine (S), threonine (T), tryptophan (W), tyrosine (Y), valine (V). Diseases: ADPKD (autosomal dominant polycystic kidney disease), ADTKD (autosomal dominant tubulointerstitial kidney disease), ADPKD (autosomal dominant polycystic kidney disease), CAKUT (congenital anomalies of the kidney and urinary tract), CKDu (chronic kidney disease of unknown etiology), dRTA (distal renal tubular acidosis), FSGS (focal segmental glomerulosclerosis), IgAN (Immunoglobulin A nephropathy), TIA (transient ischemic attack), UTI (urinary tract infection). Nucleotides: adenine (A), cytosine (C), guanine (G), thymine (T). Other: c. (nucleotide position), del. (deletion), gnomAD (the genome aggregation database), F (female), freq. (frequency), fs (frameshift), M (male), mat. (maternal inheritance), *MUC1* (Mucin 1), NA (not applicable), OMIM (online mendelian inheritance in man), p. (amino acid position), pat. (paternal inheritance), VNTR (variable number tandem repeat), VUS (variant of unknown significance). New variants are indicated with a Asterix (*) in the gnomAD frequency column.

Table S2. Logistic Regression Analysis of Patients with a Genetic Diagnosis.

Variable	OR (95% CI)	Unadjusted p-value	Benjamini-Hochberg adjusted p-value
Positive family history	2.93 (1.19, 8.05)	0.03	0.08
CKD Age 50+	0.41 (0.16, 1.02)	0.06	0.09
ESKD	1.78 (0.82, 3.86)	0.14	0.14

Footnote: Adjusted for test age and gender.

Table S3. Patient 779 Clinical Laboratory and Genetic Findings.

A. Clinical Findings

Visit	Age	Creatinine umol/L	Creatine kinase U/L	Urinalysis
First nephrology encounter	60	138	-	Trace protein, negative blood
First admission with AKI requiring RRT	61	818	3156	+1 protein, large hemoglobin
Second admission with AKI	66	651	821	+1 protein, large hemoglobin, negative erythrocyte
Third admission with AKI	67	554	22,000	+1 protein, large hemoglobin, negative erythrocyte
First kidney genetic clinic assessment	67	176	224	Trace protein, moderate hemoglobin, negative erythrocyte

B. Genetic Findings

Gene	Hg19 Pos	Transcript	Position	Mm	Gg	Xt	Dr	Ci	Ce	Dm	*In silico*	gnomAD Freq	ACMG Class.
POLG	Chr15: 89870432	NM_002693.2	c.1399G>A p. A467T	A	A	A	A	A	A	A	D	0.0005	P

A. Laboratory results from first nephrology service encounter until the kidney genetic clinic. **B.** Variant description including impact of variant on cDNA level including predicted amino acid or protein level change. **Footnote:** *In silico* prediction tools used include Polyphen-2 (http://genetics.bwh.harvard.edu/pph2), Mutation Taster (http://www.mutationtaster.org), and SIFT (http://sift.jcvi.org/); the classification D is damaging. Nucleotides (c. change): adenine (A), guanine (G). Amino acids (p. change): alanine (A), threonine (T). Evolutionary conservation of amino acid was assessed across phylogeny over 7 species: *Caenorhadbitis elegans* (Ce), *Ciona intestinalis* (Ci), *Drosophila melanogaster* (Dm), *Danio rerio* (Dr), *Gallus gallus domesticus* (Gg), *Mus musculus* (Mm), *Xenopus tropicalis* (Xt). Other abbreviations: Acute kidney injury (AKI), American College of Medical Genetics classification (ACMG, Class.), Chromosome (Chr), Genome Aggregation Database frequency (gnomAD Freq), Human genome build 19 position (Hg19 Pos), pathogenic (P), rapid response team (RRT).

Table S4. Patient 577 Clinical Laboratory and Genetic Findings.

A. Clinical Findings

Timeline	Platelet x10*9/L	Hemo-globin g/L	Creatinine umol/L	ADAMTS-13	LDH U/L	Haptoglobin g/L	Blood film for schistocyte
Prior to Transplant	104	87	514	-	-	-	NA
Post-Transplant Day-0	38	67	371	4%	408	<0.1	Mild
After 6 cycles of plasma exchange	65	71	116	-	279		-
After 1 dose of Rituximab 700mg with daily Plasma exchange	86	79	118	NA	303	-	-
On discharge post-kidney transplant	138	82	105	-	NA	-	NA
First Transplant clinic	184	90	120	8%	26	-	-
First admission with TTP relapse	101	98	134	-	290	-	-
After pulse steroid, plasma exchange and IVIG	45	86	114	-	215	-	-
After Caplacizumab (3 doses) on discharge with TTP relapse	70	88	95	37%	NA	0.86	-
Second admission with refractory TTP	24	92	106	14%	354	-	mild
After starting Eculizumab second dose on discharge	100	97	96	8%	NA	-	-
Last Transplant clinic	102	118	118	12%	341	-	-
Last Blood result	119	126	149	-	352	-	-

B. Genetic Findings

Gene	Hg19 Pos	Transcript	Position	Mm	Gg	Xt	Dr	Ci	Ce	Dm	*In Silico*	gnomAD Freq	ACMG Class.
ADAMTS13	Chr 9: 136319670	NM_ 139027.6	Hom. c.3178C>T p.R1060Y	R	K	K	K	/	/	/	C	0.0008	P
CFI	Chr4: 110682723	NM_ 000204.5	Het. c.608C>T p.T203I	G	C	/	C	/	/	/	NR	0.0005	B

A. Laboratory results tracked over time. **B.** Variant description including impact of variant on cDNA level including predicted amino acid or protein level change. **Footnote:** *In silico* prediction tools used include Polyphen-2 (http://genetics.bwh.harvard.edu/pph2), Mutation Taster (http://www.mutationtaster.org), and SIFT (http://sift.jcvi.org/); the classification C is conflicting classifications. Nucleotides (c. change): cytosine (C), thymine (T). Amino acids (p. change): arginine (R), Isoleucine (I), threonine (T), tyrosine (Y). Evolutionary conservation of amino acid was assessed across phylogeny over 7 species: *Caenorhadbitis elegans* (Ce), *Ciona intestinalis* (Ci), *Drosophila melanogaster* (Dm), *Danio rerio* (Dr), *Gallus gallus domesticus* (Gg), *Mus musculus* (Mm), *Xenopus tropicalis* (Xt). Other abbreviations: American College of Medical Genetics classification (ACMG, Class.), benign (B), Chromosome (Chr), Genome Aggregation Database frequency (gnomAD Freq), Human genome build 19 position (Hg19 Pos), Intravenous

immunoglobulin (IVIG), not applicable (NA), not reported (NR), pathogenic (P), thrombotic thrombocytopenic purpura (TTP).

Supplementary Figures

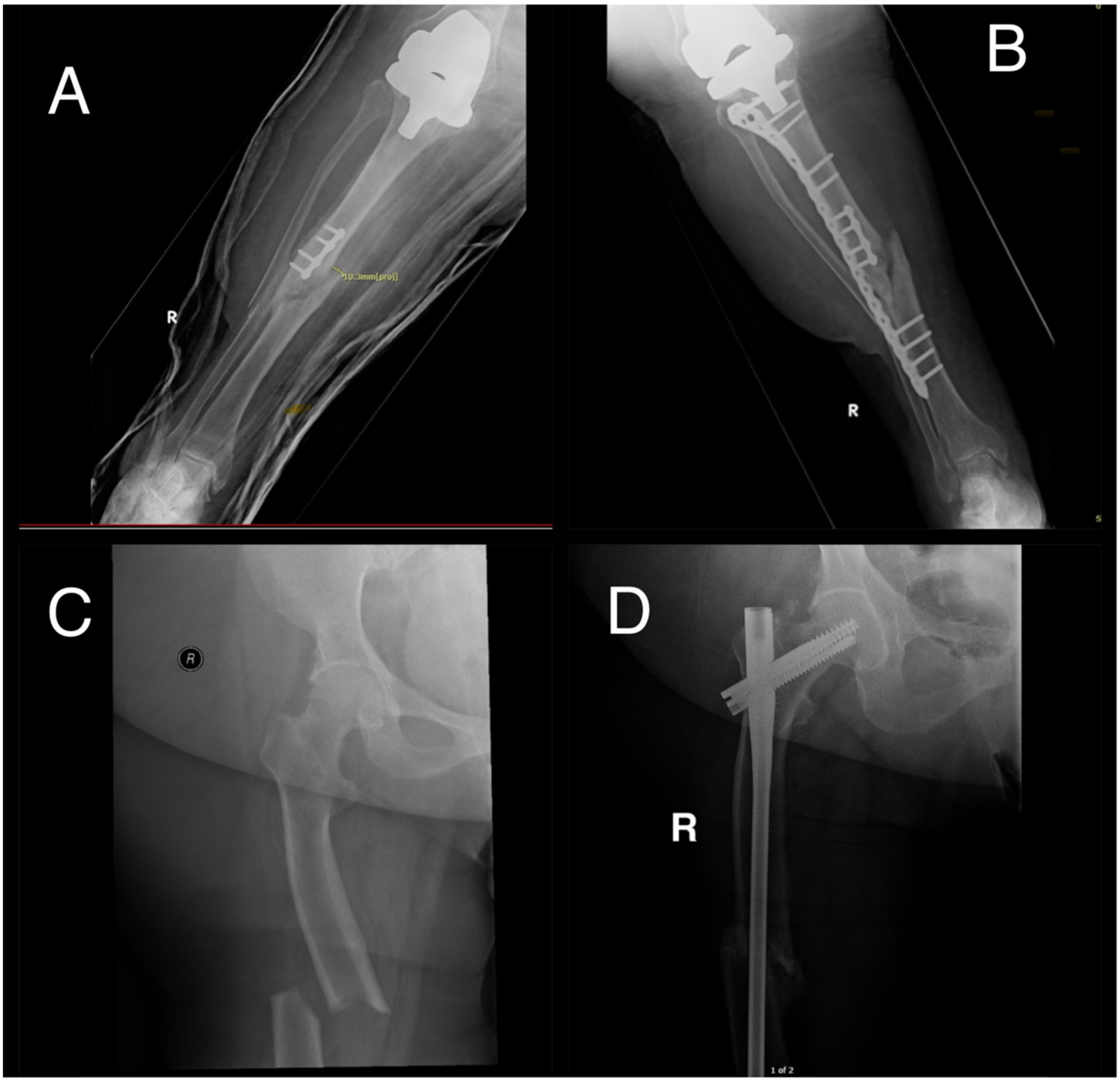

E

Genetic Findings													
Gene	Hg19 Pos	Transcript	Position	Mm	Gg	Xt	Dr	Ci	Ce	Dm	In Silico	gnomAD Freq	ACMG Class.
PHEX	ChrX: 22117263	NM_ 000444.5	c.1075_1076del p.L359Efs*20	L	L	L	L	L	L	L	NA	NR	LP

Figure S1: Patient 626 Clinical and Genetic Findings.

A: Tibial fracture is immediately distal to the mid tibial plate. **B:** Lateral plate fixation of the tibia. **C:** Transverse fracture of the mid femoral shaft. **D:** Cephalomedullary nail fixation bridging the mid shaft femoral fracture. **E:** Genetic testing results. **Note:** *In silico* prediction tools used include Polyphen-2 (http://genetics.bwh.harvard.edu/pph2), Mutation Taster (http://www.mutationtaster.org), and SIFT (http://sift.jcvi.org/). Amino acids (p. change): glutamic acid (E), leucine (L). Evolutionary conservation of amino acid was assessed across phylogeny over 7 species: *Caenorhadbitis elegans* (Ce), *Ciona intestinalis* (Ci), *Drosophila melanogaster* (Dm), *Danio rerio* (Dr), *Gallus gallus domesticus* (Gg), *Mus musculus* (Mm), *Xenopus tropicalis* (Xt). Other abbreviations: American College of Medical Genetics classification (ACMG, Class.), Chromosome (Chr), Genome Aggregation Database frequency (gnomAD Freq), Human genome build 19 position (Hg19 Pos), likely pathogenic (LP), not applicable (NA), not reported (NR).

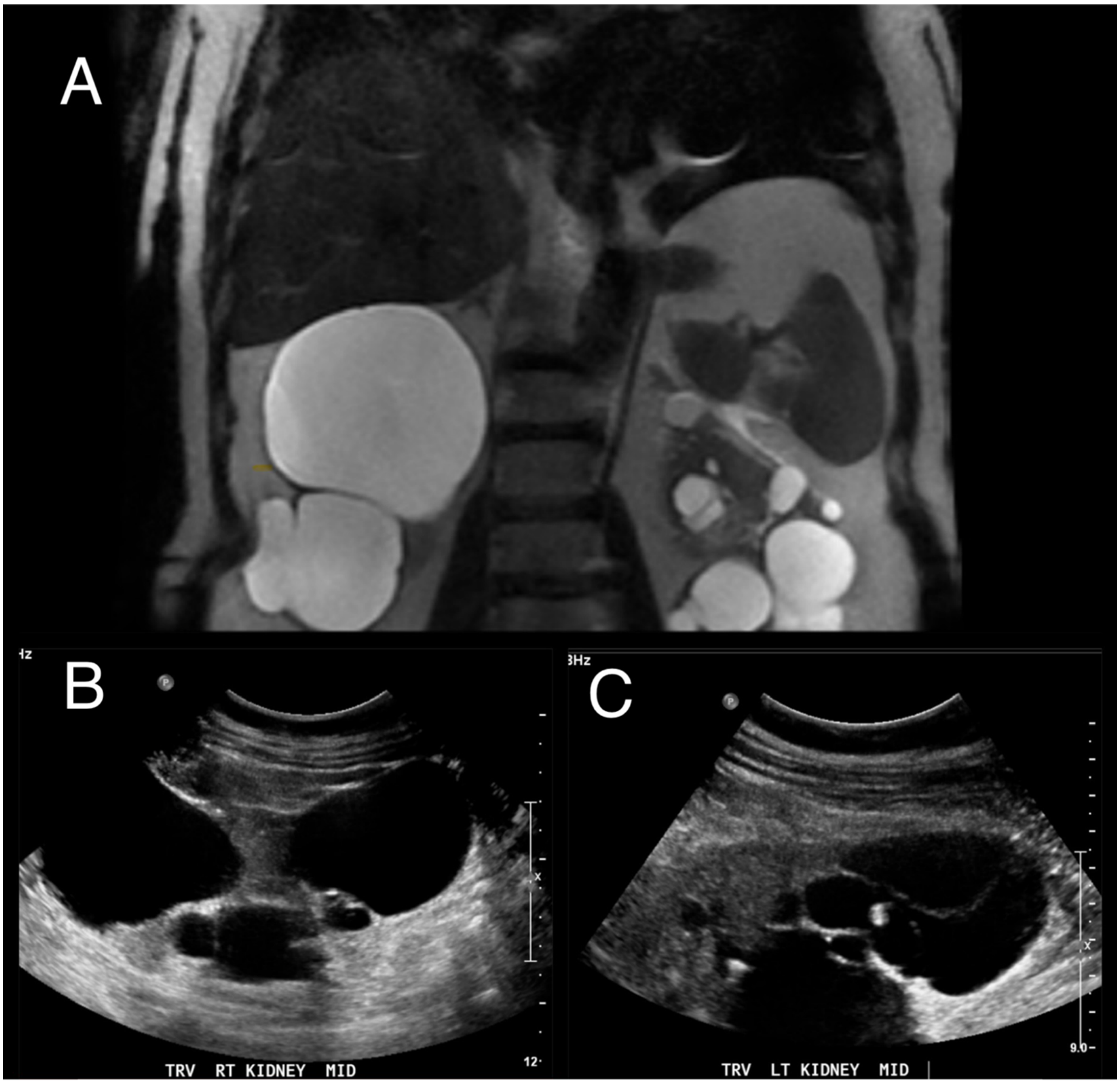

D

Genetic Findings													
Gene	Hg19 Pos	Transcript	Position	Mm	Gg	Xt	Dr	Ci	Ce	Dm	In Silico	gnomAD Freq	ACMG Class.
IFT140	Chr16: 1616192	NM_ 014714.3	c.1867_1870del, p.E623Rfs*20	Q	E	/	P	D	Q	/	NA	0.000004	P

Figure S2. Patient 518 Clinical and Genetic Findings.

A: MRI showed Multiple large cysts within the kidneys but larger on the right. **B** (right kidney) & **C** (let kidney): Ultrasound showed Innumerable bilateral renal cysts, without obvious solid lesion. The largest cyst is at the upper pole of the right kidney. **D**: Genetic testing results. **Note:** *In silico* prediction tools used include Polyphen-2 (http://genetics.bwh.harvard.edu/pph2), Mutation Taster (http://www.mutationtaster.org), and SIFT (http://sift.jcvi.org/). Amino acids: arginine (R), aspartic acid (D), glutamic acid (E), glutamine (Q), proline (P). Evolutionary conservation of amino acid was assessed across phylogeny over 7 species: *Caenorhadbitis elegans* (Ce), *Ciona intestinalis* (Ci), *Drosophila melanogaster* (Dm), *Danio rerio* (Dr), *Gallus gallus domesticus* (Gg), *Mus musculus* (Mm), *Xenopus tropicalis* (Xt). Other abbreviations: American College of Medical Genetics classification (ACMG, Class.), Chromosome (Chr), Genome Aggregation Database frequency (gnomAD Freq), Human genome build 19 position (Hg19 Pos), not applicable (NA), pathogenic (P).

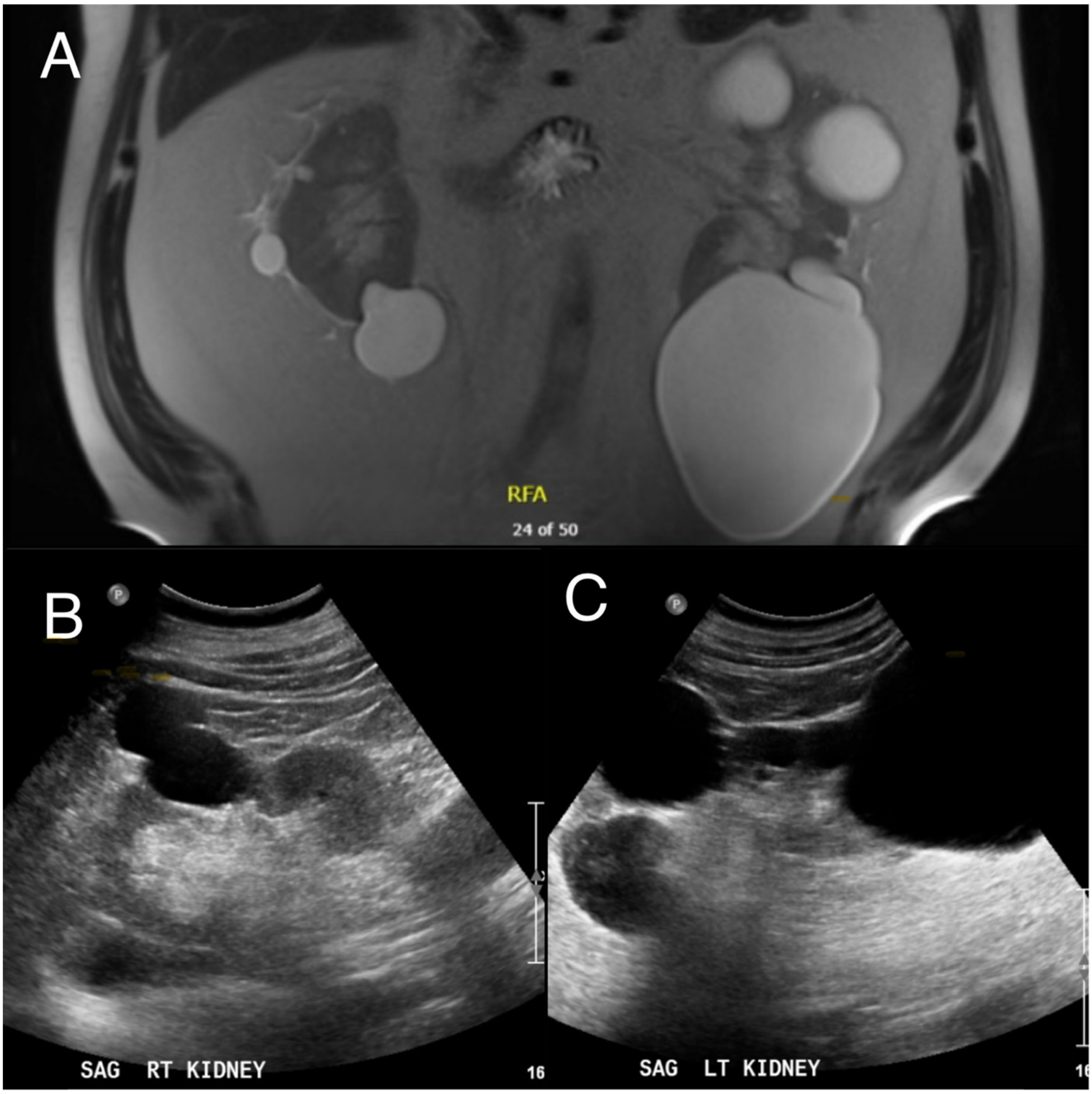

D

Genetic Findings													
Gene	Hg19 Pos	Transcript	Position	Mm	Gg	Xt	Dr	Ci	Ce	Dm	In Silico	gnomAD Freq	ACMG Class.
IFT140	Chr16: 1634215	NM_ 014714.3	c.1359_1359+3del insAC	/	/	/	/	/	/	/	NA	NR	LP

Figure S3. Patient 22 Clinical and Genetic Findings.

A: MRI showed bilateral renal cysts, the largest is in the lower pole the left kidney.
B (right kidney) & **C** (let kidney): Ultrasound showed Innumerable bilateral renal cysts, without obvious solid lesion. The largest cyst is at the lower pole of the left kidney.
D: Genetic testing results.
Note: *In silico* prediction tools used include Polyphen-2 (http://genetics.bwh.harvard.edu/pph2), Mutation Taster (http://www.mutationtaster.org), and SIFT (http://sift.jcvi.org/). Nucleotides: adenine (A), cytosine (C). Evolutionary conservation of amino acid was assessed across phylogeny over 7 species: *Caenorhadbitis elegans* (Ce), *Ciona intestinalis* (Ci), *Drosophila melanogaster* (Dm), *Danio rerio* (Dr), *Gallus gallus domesticus* (Gg), *Mus musculus* (Mm), *Xenopus tropicalis* (Xt). Other abbreviations: American College of Medical Genetics classification (ACMG, Class.), Chromosome (Chr), Genome Aggregation Database frequency (gnomAD Freq), Human genome build 19 position (Hg19 Pos), likely pathogenic (LP), not applicable (NA), not reported (NR).

Supplementary References

14. Schott C, Arnaldi M, Baker C, et al. Implementation of a kidney genetic service into the diagnostic pathway for patients with chronic kidney disease in Canada. *Kidney International Reports*. 2024;0(0). doi:10.1016/j.ekir.2024.11.004

54. Schott C, Dilliott AA, Wang J, et al. Vascular calcification in chronic kidney disease associated with pathogenic variants in ABCC6. *Gene*. 2024;927:148731. doi:10.1016/j.gene.2024.148731

55. Schott C, Colaiacovo S, Baker C, Weir MA, Connaughton DM. Reclassification of Genetic Testing Results: A Case Report Demonstrating the Need for Structured Re-Evaluation of Genetic Findings. *Can J Kidney Health Dis*. 2024;11:20543581241242562. doi:10.1177/20543581241242562
